# Pericardiocentesis vs. surgical pericardial window for first occurrence of malignancy-related pericardial effusion: a meta-analysis of retrospective studies

**DOI:** 10.1093/ehjopen/oeag018

**Published:** 2026-02-09

**Authors:** Tarek Nahle, Aditya Bhave, Karl Abou Zeid, Mohamad El Shami, Viraj R Shah, Omar M Makram, Harikrishnan Hyma Kunhiraman, Michel Abou Khalil, Manyoo A Agarwal, Nausheen Akhter, Stephanie Feldman, Arjun Ghosh, Jean-Sebastien Rachoin, Neal L Weintraub, Avirup Guha

**Affiliations:** Division of Cardiology, Department of Medicine, Medical College of Georgia at Augusta University, 1410 Laney Walker Blvd, CB-3540, Augusta, GA 30912, USA; Cardio-Oncology Program, Medical College of Georgia at Augusta University, Augusta, GA 30912, USA; Department of Medicine, Medical College of Georgia at Augusta University, Augusta, GA 30912, USA; Department of Medicine, Université Saint-Joseph de Beyrouth, Beirut, Lebanon; Department of Medicine, Corewell Health, Michigan State University, Grand Rapids, MI 49503, USA; Division of Cardiology, Department of Medicine, Medical College of Georgia at Augusta University, 1410 Laney Walker Blvd, CB-3540, Augusta, GA 30912, USA; Cardio-Oncology Program, Medical College of Georgia at Augusta University, Augusta, GA 30912, USA; Division of Cardiology, Department of Medicine, Medical College of Georgia at Augusta University, 1410 Laney Walker Blvd, CB-3540, Augusta, GA 30912, USA; Cardio-Oncology Program, Medical College of Georgia at Augusta University, Augusta, GA 30912, USA; Department of Medicine, Medical College of Georgia at Augusta University, Augusta, GA 30912, USA; Division of Cardiology, Department of Medicine, Medical College of Georgia at Augusta University, 1410 Laney Walker Blvd, CB-3540, Augusta, GA 30912, USA; Cardio-Oncology Program, Medical College of Georgia at Augusta University, Augusta, GA 30912, USA; Tulane Research Innovation for Arrhythmia Discovery (TRIAD), Cardiac Electrophysiology, Tulane University School of Medicine, New Orleans, LA 70118, USA; Cardio-Oncology Program, Cleveland Clinic Abu Dhabi, Abu Dhabi, United Arab Emirates; Division of Cardiology, Feinberg School of Medicine, Northwestern University, Chicago, IL 60611, USA; Division of Cardiology, Weill Cornell Medicine, New York, NY 10065, USA; Cardio-Oncology Services, Barts Heart Centre, St Bartholomew’s Hospital, London, UK; University College London Hospital London, London, UK; Medicine, Cooper Medical School of Rowan University, Camden, NJ 08103, USA; Division of Cardiology, Department of Medicine, Medical College of Georgia at Augusta University, 1410 Laney Walker Blvd, CB-3540, Augusta, GA 30912, USA; Cardio-Oncology Program, Medical College of Georgia at Augusta University, Augusta, GA 30912, USA; Division of Cardiology, Department of Medicine, Medical College of Georgia at Augusta University, 1410 Laney Walker Blvd, CB-3540, Augusta, GA 30912, USA; Cardio-Oncology Program, Medical College of Georgia at Augusta University, Augusta, GA 30912, USA

**Keywords:** Pericardial effusion, Cancer, Surgery, Pericardiocentesis

## Abstract

**Aims:**

Malignancy-related pericardial effusion (MRPE) is a serious complication of advanced cancer, potentially leading to life-threatening cardiac tamponade. While both pericardiocentesis and surgical pericardial window are used for management, data on comparative efficacy and safety remain heterogeneous. This study aims to compare the efficacy and safety of pericardiocentesis vs. surgical pericardial window in the management of the first occurrence of MRPE.

**Methods and results:**

We systematically searched PubMed, Cochrane, and Google Scholar up until May 2025. Articles directly comparing pericardiocentesis to surgical pericardial window, which include patients with MRPE, have first occurrence procedure outcome data, and published in English, were included. Our outcomes consisted of procedure failure (composite of inability to drain, inability to relieve symptoms, or recurrence of effusion), bleeding, infection, pneumothorax, supraventricular tachyarrhythmias (SVT), and death. A total of six articles met our inclusion criteria, representing a total of 1369 patients, 1086 of whom underwent pericardiocentesis and 253 surgical pericardial windows. Procedure failure was significantly higher in the pericardiocentesis group when compared to the surgical approach [odds ratio (OR): 2.99, 95% CI 1.10–8.14, *P* = 0.03], while more deaths were reported in the surgical pericardial window group (OR: 0.68, 95%CI 0.46–0.99, *P* = 0.05). There was no difference between the two groups for bleeding (*P* = 0.051), infection (*P* = 0.11), pneumothorax (*P* = 0.56), or SVT (*P* = 0.31).

**Conclusion:**

Both pericardiocentesis and surgical pericardial window showed comparable rates of infection, bleeding, SVT, and pneumothorax in patients with MRPE. However, due to the nature of the procedures, a lower failure rate was seen in surgical pericardial window with a higher death rate.

## Graphical Abstract

**Graphical Abstract:**
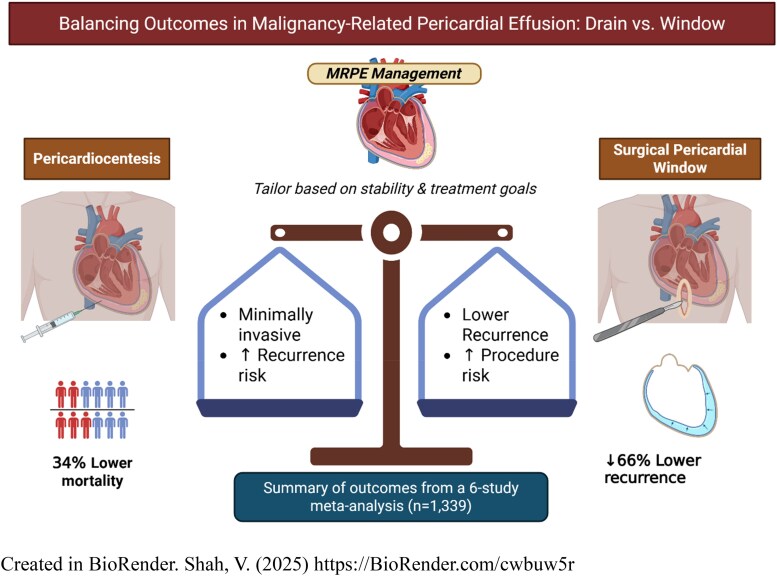

## Introduction

Malignancy-related pericardial effusion (MRPE) occurs in ∼5–15% of cancer patients, usually from metastatic seeding of the pericardium, and marks advanced disease with high morbidity and mortality.^1^ Rapid treatment is essential: unchecked fluid can quickly progress to life-threatening tamponade.^2^ Timely intervention stabilizes haemodynamics, preserves quality of life, and avoids disruption of anticancer therapy, potentially extending survival.^3^

Management centres on pericardiocentesis or a surgical pericardial window. Pericardiocentesis is often first-line because it is minimally invasive and offers swift relief; surgical window is reserved for recurrences or failed taps.^1^ Evidence on relative failure and complication rates remains conflicting, and no meta-analysis has compared the two. Our study aimed to pool existing data to clarify differences in failure and mortality between these approaches.

## Methods

Following PRISMA-2020, we performed a PROSPERO-registered search (CRD420251086930) of PubMed, Cochrane, and Google Scholar through May 2025. Search strings combined procedure terms (‘percutaneous’ OR ‘pericardiocentesis’ OR ‘pericardiostomy’ OR ‘pericardiotomy’ OR ‘drainage’ OR ‘catheter’ OR ‘sclerosis’ OR ‘Pericardial Window’), malignancy descriptors (‘malignant’ OR ‘malignancy’ OR ‘neoplastic’ OR ‘cancer’) and effusion terms (‘pericardial effusion’ OR ‘cardiac tamponade’). Comparative studies of pericardiocentesis vs. surgical window for MRPE were eligible; paediatric, non-comparative, or data-deficient reports were excluded.

**Figure 1:**
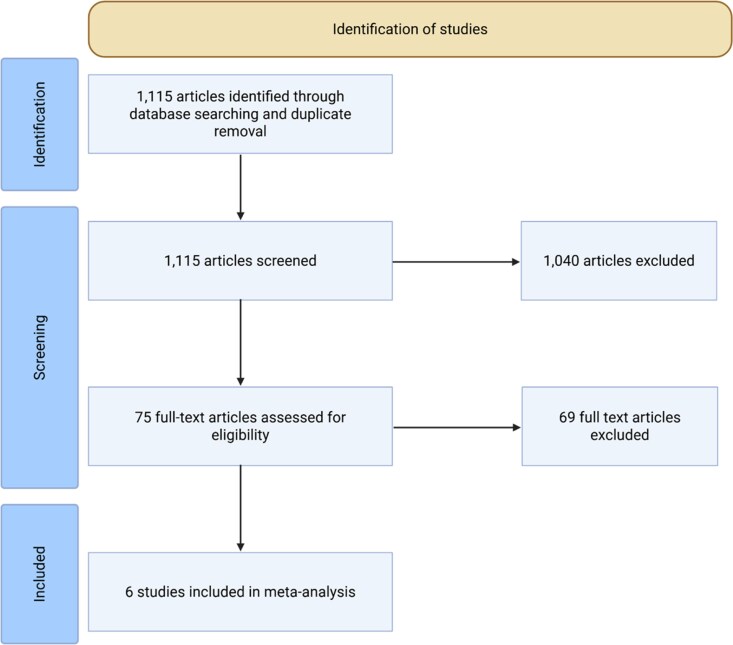
PRISMA flowchart for the article selection process.

Two reviewers independently performed screening and data extraction with a piloted form, focusing on procedural failure (composite of recurrence, inability to drain, or inability to relieve symptoms), pneumothorax, infection, bleeding, supraventricular tachyarrhythmias, and mortality at latest follow-up. Disagreements were resolved by a third reviewer. Risk of bias was assessed using the ROBINS-I tool, excluding studies with critical bias. A diagram of the selection process is shown in *Figure 1*.

Analyses used Review Manager 5.4 and R. We calculated pooled odds ratios; heterogeneity was quantified by Q, τ², and *I*². Random-effects models were applied when *P* ≤ 0.05 or *I*² > 50%; otherwise, fixed effects were used. Publication bias was explored with funnel plots; *P* ≤ 0.05 was considered significant.

## Results

**Figure 2:**
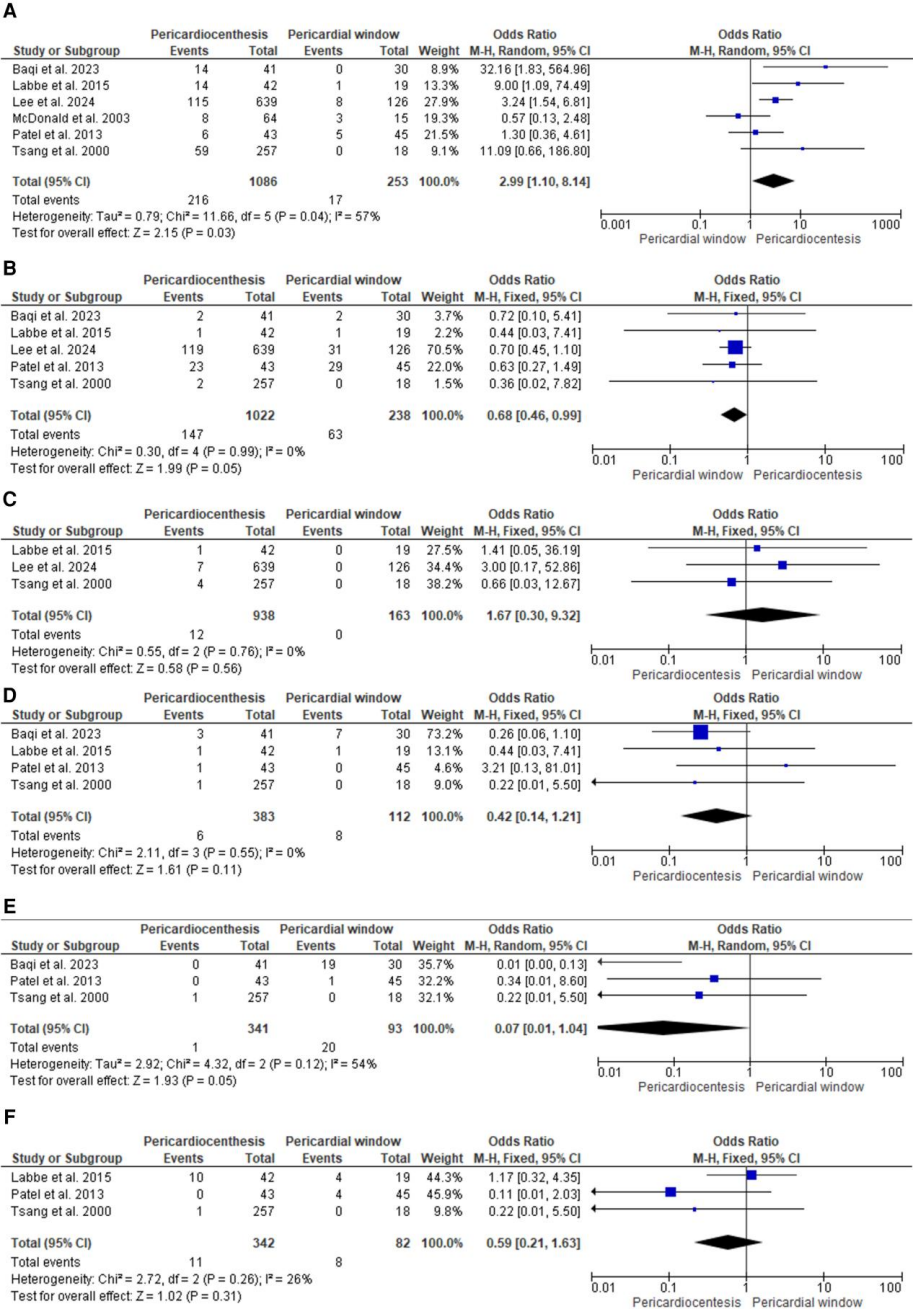
Forest plot showing the difference in (*A*) procedural failure, (*B*) death, (*C*) pneumothorax, (*D*) infection, (*E*) bleeding, and (*F*) supraventricular tachyarrhythmia between pericardiocentesis and surgical pericardial window. M-H, Mantel-Haenszel test

Six retrospective studies^4–9^ (*n* = 1369; 1086 pericardiocentesis, 253 windows; *Table 1*) met eligibility criteria. Procedure failure was higher after pericardiocentesis (OR 2.99, 95% CI 1.10–8.14; I² = 57%; *Figure 2a*). All-cause mortality favoured pericardiocentesis (OR 0.68, 0.46–0.99; I² = 0%; *Figure 2b*). No significant between-group differences were seen for pneumothorax, infection, bleeding, or supraventricular tachyarrhythmias (*Figures 2c–f*).

**Table 1:** Characteristics of the included studies

Author	Methods	MRPE total (*n*)	Pericardiocentesis *n* (mean, age)	Surgical pericardial window *n* (age)	Most common cancer	Tamponade at presentation	Volume drained
Baqi et al.^4^	Retrospective	71	41 (–)	30 (–)	Lymphoma/leukaemia total 28 (39.4%)	Total 96.1%	Total medium 67 (94.4%), total large (4 (5.6%)
Lee et al.^8^	Retrospective	765	639 (58.6)	126 (57.7)	Lung 337 P, 50 SPW	–	–
Labbé et al.^5^	Retrospective	61	42 (62 ± 11)	19 (59 ± 11)	Lung, 40 P, 12 SPW	29 P, 12 SPW	611mL ± 315 P, 498mL ± 235 SPW
Tsang et al.^7^	Retrospective	275	257 (56 ± 16)	18 (60 ± 12)	Lung, 77% P, 33% SPW	42% P, 50% SPW	64.3% large, 32.88% medium, 2.3% small in P; 67% large, 28% medium, 5% small in SPW
Patel et al.^9^	Retrospective	88	43 (61 ± 15)	45 (58 ± 12)		–	–
McDonald et al.^6^	Retrospective	79	64 (–)	15 (–)	Lung 51% total	–	–

MRPE, malignancy-related malignant effusion; P, pericardiocentesis; SPW, surgical pericardial window.

## Discussion

In this meta-analysis of malignant pericardial effusion, pericardiocentesis was associated with a higher rate of procedural failure than a surgical pericardial window, but a lower risk of post-procedural mortality. No significant differences were observed in major complication rates, including pneumothorax, infection, bleeding, or supraventricular arrhythmia.

From the included studies, Baqi et al.^4^ found that diabetes was a strong predictor of complications following the procedure, in addition to being a predictor for longer length of stay. Additionally, in their study, female patients were more likely to have more complications than male patients. However, in Labbé et al.’s study,^5^ age, sex, and the type of malignancy were not associated with higher recurrence rate, nor were the presence of an effusion at the time of cancer diagnosis, previous history of chest irradiation, presence of a tamponade at presentation, or the total volume of fluid removed.

The higher recurrence rate after pericardiocentesis is well recognized^1^; one large series found effusions reaccumulated in 18% of cases after catheter drainage vs. 6% after a surgical window.^8^ By contrast, the lower post-procedural mortality with pericardiocentesis likely reflects selection bias or perioperative risks in surgical patients, as contemporary data show no significant survival difference between the two techniques.^5^

Both procedures had low rates of major complications, indicating they are generally safe. However, our analysis is limited by retrospective study designs, potential confounding, and inconsistent reporting of patient factors (e.g. cancer type and performance status); thus, this meta-analysis is subject to the inherent limitations of retrospective research, particularly the risk of selection bias. Consequently, the potential influence of these biases on the overall findings cannot be excluded. Moreover, data on long-term outcomes, quality of life, and return to therapy were sparse, leaving uncertainty about the broader impact of each intervention. Our results should also be interpreted in the context of the moderate heterogeneity detected for some of our outcomes. This variability likely reflects differences in patient selection, disease severity, and procedural approach across retrospective studies and may contribute to the uncertainty around the effect estimates.

Future prospective studies should control for patient and disease characteristics, include longer follow-up, and report patient-centred outcomes. In summary, surgical windows provide more reliable effusion control, but pericardiocentesis remains a reasonable first-line approach. The choice of procedure should be individualized based on patient goals, clinical stability, and cancer trajectory.
